# Bromodomain proteins: protectors against endogenous DNA damage and facilitators of genome integrity

**DOI:** 10.1038/s12276-021-00673-0

**Published:** 2021-09-21

**Authors:** Seo Yun Lee, Jae Jin Kim, Kyle M. Miller

**Affiliations:** 1 Department of Molecular Biosciences, The University of Texas at Austin, Austin, TX USA; 2 Livestrong Cancer Institutes, Dell Medical School, The University of Texas at Austin, Austin, TX USA; 3 Department of Life Science and Multidisciplinary Genome Institute, Hallym University, Chuncheon, Korea

**Keywords:** DNA damage response, Stalled forks

## Abstract

Endogenous DNA damage is a major contributor to mutations, which are drivers of cancer development. Bromodomain (BRD) proteins are well-established participants in chromatin-based DNA damage response (DDR) pathways, which maintain genome integrity from cell-intrinsic and extrinsic DNA-damaging sources. BRD proteins are most well-studied as regulators of transcription, but emerging evidence has revealed their importance in other DNA-templated processes, including DNA repair and replication. How BRD proteins mechanistically protect cells from endogenous DNA damage through their participation in these pathways remains an active area of investigation. Here, we review several recent studies establishing BRD proteins as key influencers of endogenous DNA damage, including DNA–RNA hybrid (R-loops) formation during transcription and participation in replication stress responses. As endogenous DNA damage is known to contribute to several human diseases, including neurodegeneration, immunodeficiencies, cancer, and aging, the ability of BRD proteins to suppress DNA damage and mutations is likely to provide new insights into the involvement of BRD proteins in these diseases. Although many studies have focused on BRD proteins in transcription, evidence indicates that BRD proteins have emergent functions in DNA repair and genome stability and are participants in the etiology and treatment of diseases involving endogenous DNA damage.

## DNA repair: multitasking proteins

Bromodomain (BRD) proteins, known to regulate gene expression, switching particular genes on and off, also play key roles in repairing DNA damage, and studying them may help identify treatments for various diseases, including cancer. DNA damage can occur during normal cellular metabolism, for example, during copying DNA and gene expression. DNA damage is implicated in tumor formation as well as in neurodegeneration, immunodeficiency, and aging. Seo Yun Lee and colleagues at The University of Texas at Austin, USA, have reviewed new results showing how BRD proteins function in repairing DNA damage. They report that when DNA is damaged during copying in BRD-deficient cells, tumors can result. They also report that defects in BRD proteins are often present in cancers. Studying how BRD proteins function in both healthy and diseased cells could help to identify new therapies.

## Introduction

It has been estimated that the genome within each individual human cell is subjected to tens of thousands of DNA lesions per day^1,2^. The origins of the DNA damage that cause these lesions, which include base damage, intra- and interstrand cross-links, DNA–protein cross-links, and single- and double-strand breaks (SSBs/DSBs), can come from both endogenous and exogenous sources^3^. The most frequent DNA lesions result from base damage and SSBs, whereas DSBs are rarer; their frequency is estimated to be ~1 per cell per hour^2,4^. DSBs are considered to be one of the most deleterious DNA lesions for genome integrity owing to their potential for generating mutations and chromosomal aberrations^1^.

DSBs can be generated by exogenous stimuli such as ionizing radiation and chemicals, as well as internal cellular processes such as metabolism, errors in DNA replication, and/or transcription^2^. To suppress deleterious outcomes from DSBs, cells employ two major DSB repair pathways called homologous recombination (HR) and nonhomologous end-joining (NHEJ). The HR pathway is high fidelity and considered error-free because it engages a homologous sequence, for example, a sister chromatid, as a template to repair the break. NHEJ, on the other hand, is a more error-prone repair pathway because it ligates DNA break ends together without the use of a template. DNA damage response (DDR) pathways coordinate these repair pathways during different phases of the cell cycle. This enables cells to arrest the cell cycle to allow efficient DNA repair and to engage the correct pathway in the appropriate cell cycle phase. For example, HR is prevalent in the S/G2 phase of the cell cycle, where sister chromatids are available as templates for the repair of DSBs. The DDR also triggers cell death or senescence in cells if DNA damage is insurmountable, allowing the DDR to function in cell fate decisions and cancer suppression pathways^5^.

Genome instability is an important contributor to tumorigenesis in many human cancers, and this contribution is mediated, for example, by the accumulation of genetic alterations ranging from single-nucleotide mutations to chromosome rearrangements that can predispose cells toward malignant transformation. Recent large-scale genome sequencing studies, including those in the International Cancer Genome Consortium (ICGC) portal and The Cancer Genome Atlas (TCGA), have identified mutations in DNA repair genes, tumor suppressor genes, and oncogenes that are key driver mutations associated with different types of cancers^6,7^. The Pan-Cancer Analysis of Whole Genome (PCAWG) Consortium study of the ICGC and TCGA characterized mutational signatures in most types of cancers. For example, they identified 49 single-base substitutions, 11 doublet-base substitutions, 4 clustered-base substitutions, and 17 small insertion-and-deletion signatures. These analyses revealed that the overlapping mutational signatures are potentially generated by multiple processes involving DNA replication, transcription, and other DNA-damaging processes that occur within cells and are actively being identified and characterized^8^. For example, mutational signatures have been identified in BRCA1- and BRCA2-mutant tumors and the signatures themselves can predict HR deficiency^9^. Assessment of mutation burden through whole-genome sequencing of tumors or CRISPR screens of putative mutation mitigators has also revealed many other contributing factors, including mismatch repair deficiencies, replication, carcinogens, and germline mutations^10,11^.

**Fig. 1:**
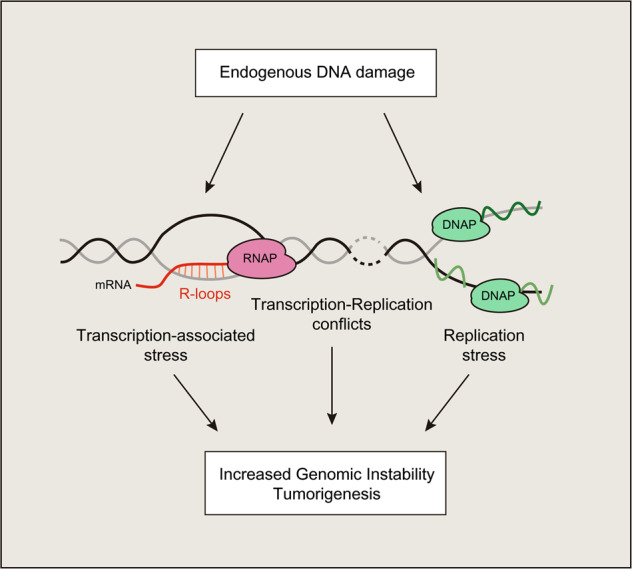
Endogenous DNA damage contributes to genome instability and tumorigenesis. Formation of endogenous DNA damages can be generated through several mechanisms. These include transcription-associated stress involving R-loops, transcription-replication conflicts, and defects in replication.

Chromatin consists of histone proteins arranged into nucleosomes that function to organize the genome, which regulates chromatin structure and function. Histones, the basic building blocks of chromatin, are modified by posttranslational modifications (PTMs), including acetylation, ubiquitination, and methylation^12^. Bromodomains (BRDs) are acetyl-lysine binding motifs that are found in 42 proteins in mammalian cells^13^ and include ATP-dependent chromatin remodelers, histone acetyltransferase (HAT)-containing complexes, and transcriptional regulators. Through their ability to recognize acetylation signals on chromatin via their BRDs, these proteins play important roles in regulating gene expression and DNA repair processes^14,15^. In this review, we highlight recent work that establishes new functions for BRD proteins in endogenous DNA damage suppression, with a focus on transcription-mediated DNA damage and replication stress responses (Fig. 1). Understanding how BRD proteins regulate transcription/replication-associated DNA damage is significant given the involvement of these pathways and DNA damage in human diseases. BRD proteins represent current drug targets in oncology; for example, BET inhibitors target a class of BRD proteins and are currently in preclinical and clinical studies for use in cancer^16–19^. New insights revealing how BRD proteins contribute to the etiology and potential suppression of DNA damage-associated human diseases may deliver a better understanding of these diseases and new therapeutic strategies targeting these chromatin reader BRD proteins.

## Replication stress

The faithful duplication and transmission of genetic information are vital for maintaining genome stability from one generation to the next in eukaryotic cells^20^. Given that human diploid cells must replicate over six billion DNA bases every cell division, defects in this process are known to contribute to an increased risk for human cancer^21–23^. A prime example comes from studies in colorectal cancer where defects in mismatch repair genes result in microsatellite instability and increased cancer risk not only for colorectal cancer but also for other cancers^24,25^. Defects in the replicative polymerases themselves are also found in cancers and are associated with hypermutation^26^. Replication stress is a major contributor to genome instability through several mechanisms involving DNA lesions, abnormal DNA structures, conflicts with transcription, or the depletion of nucleotide pools, which results in slowing, stalling, or breakage of replication forks^22,27,28^. In this section, we describe how BRD proteins regulate replication processes to protect human cells from endogenous DNA damage and genome instability.

## BAZ1B

**Fig. 2:**
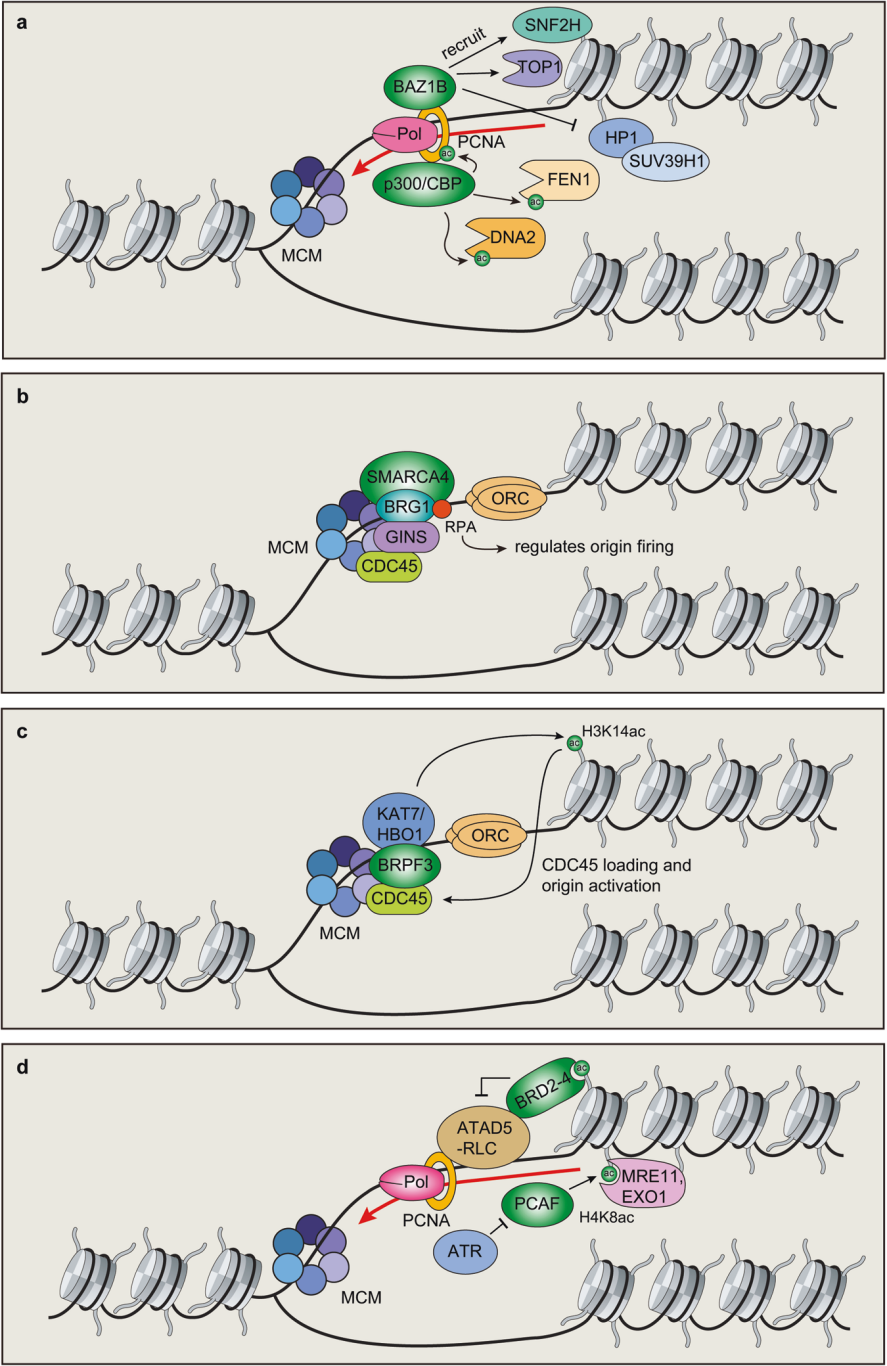
Bromodomain (BRD) proteins function in replication stress responses. **a** BAZ1B recruits SNF2H and topoisomerase I (TOP1) to replication forks, which inhibits HP1/SUV39H1-mediated H3 methylation and heterochromatin. p300/CBP acetylates PCNA, FEN1, and DNA2, which promote genome stability during replication. **b** SMARCA4/BRG1 regulates origin firing to protect genome stability. **c** BRPF3/HBO1 regulates origin activation by promoting H3K14ac and CDC45 loading. **d** BET proteins regulate PCNA levels at replication forks by regulating ATAD5 activity. PCAF acetylates H4K8 to recruit MRE11/EXO1 to stalled replication forks in BRCA-deficient cells. PCAF activity at stalled forks is limited by ATR phosphorylation.

Several BRD proteins have been demonstrated to be involved in the replication. The BRD protein BRD adjacent to zinc finger domain, 1B (BAZ1B)/Williams syndrome transcription factor (WSTF) is a core component of the WICH complex, a chromatin-remodeling complex that mobilizes nucleosomes to maintain a regular nucleosome structure. BAZ1B localizes to replication forks through direct interaction with the replication processivity factor PCNA^29^. BAZ1B helps to maintain an open chromatin structure during replication. BAZ1B does not function alone but rather recruits SNF2H (SMARCA5), which is an ATP-dependent chromatin-remodeling factor. Depletion of BAZ1B results in increased compaction of newly replicated chromatin and a concomitant increase in the accumulation of heterochromatin marks, including H3K9me3 and H3K27me2^29^. The alteration of chromatin structure in BAZ1B-deficient cells is associated not only with aberrant heterochromatin histone modifications but also with nonhistone proteins, including HP1α and HP1β. These effects were specific to cells in S-phase, suggesting that BAZ1B functions to inhibit heterochromatin formation during replication and that loss of BAZ1B or SNF2 resulted in ectopic heterochromatin formation (Fig. 2a). Although BAZ1B KO cells display unaltered cell cycle distribution^30^, SMARCA5 deficiency leads to reduced replication fork progression^31^. Whether increased heterochromatin formation in cells deficient in BAZ1B and/or SMARCA5 is the root cause of replication stress in these cells is unknown, and further analyses will be required to determine the involvement of aberrant heterochromatin formation and replication stress responses in cells lacking these chromatin-remodeling complexes.

In an unbiased proteomic analysis using an isolation of proteins on nascent DNA (iPOND) approach, BAZ1B was detected on replicating DNA following treatment with camptothecin (CPT), a topoisomerase I (TOP1) inhibitor that stalls replication forks^30^ through its ability to trap TOP1 on DNA and block the TOP1 cleavage/ligation cycle^32^. These TOP1-DNA cleavage complexes (TOP1ccs) result in replication-mediated DSBs^33^. Ribeyre et al.^30^ found that the BAZ1B-SMARCA5 complex recruits TOP1 to replication forks. Depletion of BAZ1B results in tolerance to CPT-induced DSBs due to reduced TOP1 loading onto replication forks. During normal replication, BAZ1B-SMARCA5 appears to promote replication fork progression by recruiting TOP1 to forks to resolve torsional stress that is associated with replication (Fig. 2a). Thus, the BAZ1B BRD chromatin-remodeling protein has several functions during replication that are required to avoid replication stress.

## HATs p300 and CBP

The highly related HATs p300 and CREB-binding protein (CBP) are transcriptional coactivators involved in signal transduction pathways that regulate multiple cellular processes, including cell growth, differentiation, cell cycle progression, and cell death^34–36^. In addition, p300/CBP has been shown to directly bind to ATR kinase, which is required for CHK1 activation. Loss of p300/CBP results in defects in the intra-S phase checkpoint and replication^37^. p300/CBP acetylates several replication-associated proteins involved in the protection of genome stability. For example, Cazzalini et al.^38^ observed that p300/CBP acetylates PCNA, which has many functions, including acting as a molecular platform for recruiting numerous DNA replication proteins to the replication fork^39^. PCNA is an essential protein requiring tight regulation to maintain genome stability during DNA replication, as it orchestrates multiple DNA replication processes through its scaffolding function^40,41^. p300/CBP interacts with PCNA through a C-terminal transactivation domain and acetylates PCNA, with CBP displaying increased activity toward PCNA compared with p300. These acetylation marks on PCNA promote the removal of chromatin-bound PCNA, which is degraded upon DNA damage after ultraviolet light (UV) exposure. Four p300/CBP-mediated acetylation sites on PCNA were identified by mass spectrometry analysis, and site-specific mutation of these sites was shown to prevent PCNA release from DNA damage sites by blocking ubiquitination-mediated proteasomal degradation^38^. These mutations also led to impaired DNA replication and repair synthesis, indicating that p300 and CBP regulation of PCNA levels is required for genome stability during both normal replication and following DNA damage by UV radiation.

Flap endonuclease I (FEN1) and DNA2 endonuclease/helicase (DNA2) are also acetylated by p300^42,43^. FEN1 and DNA2 sequentially coordinate their nuclease activities for efficient resolution of flap structures that are created during the maturation of Okazaki fragments and repair of DNA damage. p300-mediated acetylation differentially regulates FEN1 and DNA2 functions. The acetylation of FEN1 by p300 suppresses its endonuclease activity^42^, whereas the acetylation of DNA2 promotes its nuclease and helicase activities^43^. Inhibition of FEN1 activity by acetylation after UV damage may trigger the error-free repair system mediated by HR. Acetylation of DNA2 by p300 significantly increases the binding efficiency of DNA2 to DNA substrates^43^. The p300-mediated acetylation of FEN1 and DNA2 increases DNA2 activity with concomitant FEN1 inhibition, thereby enhancing the processing of longer flaps. At sites of replication, this regulation might be more effective for the removal of incorrect bases possibly introduced by error-prone DNA Pol alpha during DNA repair. Given the functions of p300/CBP acetylated substrates, including FEN1, DNA2, and PCNA, during replication, these findings highlight the important role that p300/CBP plays in protecting genome stability during replication as well as after DNA damage events that involve DNA synthesis, including UV damage (Fig. 2a).

## MLL1/2

Myeloid/lymphoid or mixed-lineage leukemia (MLL) is a histone methyltransferase involved in transcription during early development and hematopoiesis^44–46^. The MLL protein catalyzes H3K4 methylation through its SET domain, which mediates chromatin modifications for epigenetic transcriptional activation^47^. The levels of MLL protein are controlled differently during the cell cycle and are regulated by SCF^skp2^ E3 ligase and APC^cdc20^ at the S and M phases^45,48,49^. In the normal S phase, MLL is ubiquitinated and degraded by the SCF^skp2^ E3 ligase; however, the interaction between MLL and the SCF^skp2^ E3 ligase is disrupted by genotoxic stress, including hydroxyurea (HU) treatment, which accumulates cells in S phase due to the depletion of nucleotide pools. Liu H et al.^50^ showed that MLL acts during the mammalian S-phase checkpoint response through phosphorylation by the checkpoint kinase ATR. Indeed, MLL is phosphorylated at serine 516 by ATR, which disrupts the interaction between MLL and the SCF^skp2^ E3 ligase, leading to the accumulation of MLL in S phase. The accumulated MLL methylates histone H3K4 at late replication origins and inhibits the loading of CDC45. This MLL-mediated inhibition delays DNA replication, which is required to coordinate DNA repair and replication progression. The functional importance of these observations is supported by observations made in MLL-deficient cells, which display defective DNA synthesis following DNA damage and chromatid-type chromosomal aberrations, phenotypes consistent with MLL functioning in replication and S-phase checkpoint control. How the BRD domain within MLL contributes to these replication-associated functions is unknown. Given that MLL fusions are found in mixed-lineage leukemia and that these fusions remove the BRD domain^51^, it is tempting to speculate that this domain, along with other chromatin reader domains affected by MLL fusions, may contribute to replication stress and leukemogenesis.

## SMARCA4/BRG1

BRG1/SMARCA4 is a catalytic subunit of the SWI/SNF ATP-dependent chromatin-remodeling complex^52,53^. BRG1 was observed to colocalize with origin recognition complexes, GINS complexes, and PCNA using extended chromatin fiber analysis^54^ (Fig. 2b). BRG1 mutant mouse embryos and knockdown cells display growth defects and a decline in cell proliferation, which may be caused by a reduction in replication fork progression, which was observed in these cells using DNA fiber assays^54^. BRG1 interacts with TOPBP1^54,55^, a replication stress response factor, and RB, a cell cycle inhibitor whose loss results in an aberrant S-phase checkpoint response to DNA damage^56^. Thus, several potential mechanisms involving BRG1 activity within the SWI/SNF complex can be envisioned concerning how this factor supports replication. It has also been reported that BRG1-mediated chromatin remodeling is critical for maintaining genome stability to prevent cancer. In nonsmall cell lung cancers (NSCLCs), BRG1 is frequently inactivated, and deletion of BRG1 leads to replication stress by unregulated origin firing^57^. In eukaryotic cells, replication origin firing proceeds from the formation of a prelicensing complex protein that includes ORC1–6, CDC6, CDT1, and MCM2–7 proteins. After the prelicensing complex is assembled, origins are activated by S-phase kinases^58,59^. In BRG1-deficient cells, origin firing was observed to increase, and mass spectrometry analyses identified the single-strand binding protein RPA as an interaction partner with the SWI/SNF complex^57^. Unregulated origin firing has been shown to promote replication fork defects and increase chromosomal breakage^60^. In the case of BRG1 deficiency in lung cancer cells, these cells were shown to be sensitive to ATR inhibitors, which may provide a therapeutic strategy for targeting tumors deficient in the BRD protein BRG1 or other potential loss-of-function mutations that may be present in other SWI/SNF protein complex members^53,61^. These results highlight how BRG1 and associated SWI/SNF complex partner proteins regulate replication processes that are involved in the suppression of both replication stress and tumorigenesis, functions that may ultimately be linked.

## BRPF3

Origin hyperactivation by oncogenic signaling contributes to genome instability and tumorigenesis^62–64^. Mutation of the retinoblastoma/E2F pathway or dysregulation of CDK activity leads to perturbation of licensing or initiation, which in turn causes the unscheduled firing of origins^64^. When dysregulated, the oncogene RAS can induce a hyperproliferation phase accompanied by increased origin firing^65^. Thus, tight regulation of replication initiation is vital for maintaining genome integrity to prevent tumorigenesis^62^. BRD and PHD finger containing 3 (BRFP3) is a scaffold protein for various HAT proteins, including MOZ/MORF and the HBO1 complex^66–68^, with the KAT7/HBO1-BRFP3-containing complex acetylating histone H3K14 (Fig. 2c). Feng et al.^67^ showed that BRPF3/HBO1 promotes H3K14 acetylation at select replication origins and that loss of BRPF3 resulted in reduced origin activation. BRPF3, HBO1, and H3K14ac were found to accumulate at active replication origins and upon replication stress. BRPF3-deficient cells displayed reduced DNA damage signaling and increased replication following release from HU-treated cells. These results suggest that the BRD protein BRPF3 regulates origin firing (Fig. 2c), which may impede recovery upon replication stress. Replication stress is known to reduce histone acetylation^69^, and these effects are likely to also impact chromatin recognition and recruitment of BRD proteins. How acetylation at the replication fork impacts BRPF3 and its chromatin localization through BRD reader functions is unknown. Alterations in acetylation during replication and stress responses are likely to affect this and potentially other BRD proteins, a question warranting future investigations.

## BET proteins

Bromodomain and ExtraTerminal motif (BET) proteins, which consist of BRD2, BRD3, BRD4, and BRDT, harbor two BRDs and one extraterminal (ET) domain. The two BRDs recognize acetylated histones, and the ET domain interacts with various other proteins to regulate transcription and DNA repair^70^. Large-scale quantitative mass spectrometry analyses revealed that several BET proteins are recruited to replication forks^71^. Two studies identified that BET proteins, including BRD2, BRD3, and BRD4, interact with ATAD5^71,72^, a factor that promotes PCNA sliding clamp unloading from DNA^73–76^. PCNA loading and unloading are critical for efficient DNA replication/repair. If PCNA unloading is not properly controlled, DNA replication can be prematurely terminated, resulting in genome instability. Wessel et al.^71^ determined that BRD3 interacts with ATAD5 through its ET domain and inhibits the ATAD5 complex to control PCNA levels on chromatin. Mapping of ATAD5 interaction regions with BET proteins identified an ET domain-binding motif spanning amino acids 596–692 within ATAD5, which mediated the interactions with BRD2, BRD3, and BRD4. Interestingly, the ATAD5–BRD4 complex coimmunoprecipitated acetylated histones, including H4K5ac (Fig. 2d)^72^. An inability of BRD4 to interact with ATAD5 would result in increased ATAD5 association with PCNA and a reduction in PCNA loading. Treatment with the BET inhibitor JQ1 reduced the interaction between ATAD5–BRD4 and acetylated histones on chromatin, suggesting that BET inhibition is likely to diminish replication in part by inhibiting the binding of ATAD5–BRD4 to chromatin. Defects in DNA replication initiation resulting from JQ1 treatment have also been reported, a defect linked to regulation of the prereplication factor CDC6 by BRD4^77^. BRD2 and BRD4 were also found to interact with TICRR/TRESLIN, another protein required for DNA replication initiation, which helps explain the requirement for BET proteins for DNA replication^78^. Interestingly, increased cancer cell killing was observed in JQ1- and ATRi-treated cells, consistent with replication stress being present in cells deficient for BET proteins. Therefore, transcriptional regulators of the BET family play additional roles during replication that are required to maintain genome integrity but that, if deficient, may represent therapeutic strategies using replication stress-targeting drugs, including ATR inhibitors.

## PCAF

DNA damage during S-phase must be repaired to avoid replication stress, which, if left unchecked, can result in stalled, collapsed, broken, and degraded replication forks. To prevent these dangers to the replication fork apparatus, stalled replication forks can reverse and undergo branch migration in the direction opposite to that of the progressing fork. These activities on the fork are catalyzed by several DNA translocases, including ZRANB3, HLTF, and SMARCAL1^79–82^. These proteins are able to promote fork reversal and the formation of a “chicken foot” structure that protects forks from degradation through the loading of RAD51 by BRCA1 and BRCA2^83,84^. Fork reversal is a protective mechanism that ensures fork stabilization, prevents collapse, and promotes fork restart. In BRCA-deficient cells, PARP inhibitor-induced DNA damage generates stalled and degraded replication forks. Endonucleases are able to degrade replication forks in these cells because RAD51 cannot be loaded onto the reversed fork to protect nascent DNA. Treatment of BRCA-deficient cells with PARPi results in cell death through fork degradation, highlighting how replication fork stability is a critical factor for PARP inhibitor responses in BRCA-deficient cancers^85^.

In recent work, p300/CBP-associated factor (PCAF) was identified as a regulator of replication fork stability in BRCA-deficient cells^86^. PCAF, also known as K (lysine) acetyltransferase 2B (KAT2B), has BRD and HAT activity. PCAF was found to acetylate histone H4K8, which facilitated the recruitment of the nucleases MRE11 and EXO1 to stalled replication forks in BRCA1- and BRCA2-deficient cells. Biochemical assays with purified proteins and modified peptides revealed that MRE11 and EXO1 likely bind to this histone mark, despite the lack of any discernable acetyl-lysine binding motif. In BRCA-deficient cells, MRE11 and EXO1 degrade stalled replication forks, which leads to replication fork degradation and sensitivity to PARP inhibitor treatment^85,87–89^. Interestingly, PCAF levels were found to be reduced in several BRCA-deficient breast cancer cell lines and BRCA2-mutant breast cancer tumors. Depletion of PCAF in BRCA1- or BRCA2-deficient cancer cells resulted in resistance to PARPi and stabilization of stalled replication forks^86^. Recruitment of PCAF to stalled replication forks still occurred in PCAF mutants lacking either the HAT or BRD domains. Engagement of stalled forks by PCAF was mapped to the N-terminus of PCAF (amino acids 1–320). However, HAT activity of PCAF was required to promote fork degradation, consistent with H4K8ac mediating the interaction between MRE11 and EXO1 and the reversed forks that occur in BRCA-deficient cells upon replication stress. Taken together, these findings indicate that PCAF is a critical chromatin-modifying factor involved in replication fork stability and PARP inhibitor sensitivity in BRCA-deficient cells^86,90^. It cannot be ruled out, however, that PCAF may also perform functions during replication and stress responses in BRCA-proficient cells.

## Transcription–replication conflicts

Transcription involves the movement of large, multiprotein molecular machines of RNA polymerases that synthesize RNA from the DNA template. The movement of RNA polymerases occurs on the same DNA template as replication machinery and, when encountered, can result in a conflict resulting in endogenous DNA damage, a potential source of genome instability in many cancers^91^. During transcription, RNAs can bind to template DNA to generate a three-stranded DNA–RNA hybrid known as an R-loop (Fig. 1). An inability to resolve R-loops results in an inhibition of transcription, which itself can increase the incidence of conflicts with replication. To prevent R-loop formation and accumulation, cells express several R-loop regulators that include helicases, topoisomerase, and RNase H enzymes that are capable of processing and removing R-loops during transcription^92–95^. Given the well-known involvement of BRD proteins in transcription, it is perhaps not too surprising that BRD proteins also play a role in transcription–replication conflicts and R-loop suppression and/or resolution in mammalian cells.

## BET proteins

**Fig. 3:**
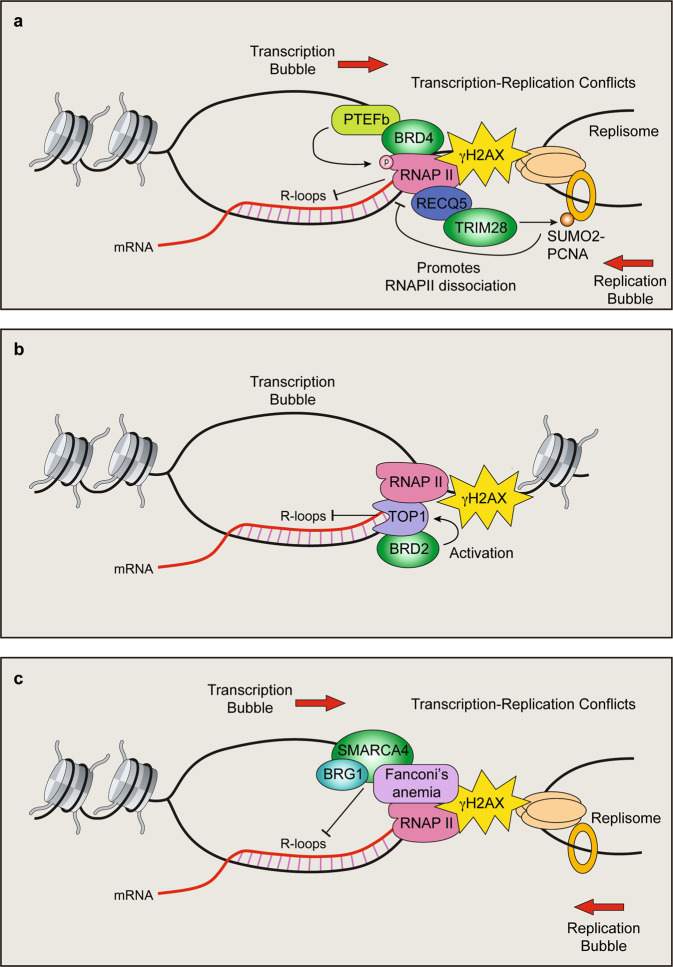
The role of bromodomain (BRD) proteins in transcription-mediated DNA damage. **a** BRD4/P-TEFb-mediated RNAPII phosphorylation and TRIM28-mediated PCNA SUMOylation coordinate transcription and replication to avoid R-loop-mediated conflicts. **b** BRD2 promotes topoisomerase I (TOP1) activity to suppress R-loop formation and DNA damage. **c** SMARCA4/BRG1 suppresses R-loop formation to limit transcription–replication conflicts in a pathway epistatic with the Fanconi anemia repair factor FANCD2. γH2AX indicates DNA breaks.

The BET proteins BRD2, BRD3, BRD4, and BRDT are regulators of transcription, including global transcription elongation^96^. For example, BRD4 recruits and activates the positive transcription elongation Factor b, P-TEFb. BRD4 interacts with P-TEFb to release it from the inactive complex that contains 7SK-snRP (7SK RNA, HEXIM1, LARP7, and MEPCE)^97,98^ (Fig. 3a). BRD4 stimulates the kinase activity of P-TEFb by promoting the phosphorylation of the C-terminal domain (CTD) of RNA polymerase II^97^. Interestingly, inhibition of BET proteins either by siRNA or the use of small-molecule inhibitors results in R-loop formation and DNA DSBs^14,99,100^. A comprehensive BRD proteomic approach revealed that BRD2 directly interacts with TOP1. TOP1 relaxes torsional stress within DNA by transiently breaking one of the two strands of DNA, relaxing the strand before reannealing the SSB^101^. TOP1 is known to function on R-loops and to remove torsional stress that builds up during transcription^102,103^. Using purified proteins, BRD2 was found to directly promote TOP1 activity via a region mapped to the C-terminus. In BRD2-depleted cells, TOP1 activity was likely diminished, explaining the increased R-loop formation that was observed in BET-inhibited cells (Fig. 3b). It was also found that in BRD2-deficient cells, topoisomerase II (TOP2) generates DSBs. These results suggest that BET inhibition can result in DSB formation through aberrant R-loop formation, which may be relevant to the use of BET inhibitors in the clinic.

Transcription and replication conflicts can trigger DSBs, leading to genome instability (reviewed in ^91,104^). BET inhibition also induces transcription and replication conflicts^99,100,105^. BRD4 loss generates an increase in R-loops, resulting in DNA damage owing to the collision of these structures with the replication machinery^100^. R-loops were found to accumulate within BRD4, JMJD6, and CHD4 coregulated genes. Interestingly, the DDR protein TOPBP1, which is required for activation of the ATR-CHK1 pathway, was transcriptionally suppressed by BRD4 inhibition. The increased damage owing to transcription–replication conflicts resulted in replication stress, and these cells were unable to mount an effective replication stress response owing to the additional effect of depleting TOPBP1 that resulted in cell death. BRD4 inhibition appears to kill cancer cells through multiple mechanisms, including increased transcription–replication induced DNA damage and suppression of the ATR pathway. In another study, loss of BRD4 was also shown to induce R-loop formation and DNA damage^99^. Here, BRD4 deficiency was shown to induce pausing of RNA polymerase II pausing on BRD4-occupied chromatin, which caused R-loop formation and transcription–replication conflicts^99^. BRD4 interactions with P-TEFB through a CTD were required to suppress R-loop formation, supporting the involvement of RNA Pol II elongation defects in transcription–replication conflict formation. Consistent with these results, BET inhibition by either JQ1 treatment or BRD4 depletion resulted in an overall increase in RNA synthesis^105^. This was accompanied by a reduction in replication fork speed that occurred in a transcription-dependent manner, suggesting the presence of transcription–replication conflicts. Of note, these authors found that the HR protein RAD51 and the P-TEFB inhibitor HEXM1 protected cells from BRD4 deficiency-mediated replication fork slowing induced by DNA damage. Taken together, these studies highlight the complex relationship between replication and transcription, which must be managed to suppress any potential conflicts that can result in aberrant transcription and replication responses. Failure to do so can lead to DNA damage and altered transcriptional regulation that collectively can trigger genome and epigenome instability.

## TRIM28

SUMOylation is a PTM that is known to be involved in the regulation of PCNA and replication^25,106,107^. For example, SUMO1-conjugated PCNA recruits the PARI helicase to restrict unscheduled HR at replication forks^108,109^. Unscheduled recombination at replication forks can generate DSBs and sister chromatid exchanges. PARI suppresses HR by suppressing the formation of RAD51-DNA structures during replication. In addition to SUMO1, the BRD protein TRIM28 catalyzes SUMO2 conjugation onto PCNA^110^. In addition to containing a BRD within its C-terminus, TRIM28 harbors an N-terminal RING domain, which has ubiquitin and SUMO E3 ligase activity. RECQ5 and RNAPII complex proteins regulate SUMOylation of PCNA, which suppresses the formation of transcription–replication conflicts^111–117^. TRIM28 contains a PIP (PCNA-interacting protein) motif that is found with its BRD and cooperates with RECQ5 for SUMO2 conjugation of PCNA^110^ (Fig. 3a). This activity antagonizes SUMO1 conjugation of PCNA and is required to avoid transcription–replication conflict-induced DNA breaks.

Conflicts between transcription and replication are major contributors to DNA breaks at common fragile sites (CFSs) that are responsible for the fragility of these genomic loci^118–120^. CFSs are hotspots for genomic rearrangements and mutations in cancers, which are often associated with the deletion of tumor suppressor genes and the amplification of oncogenes^121–123^. The stability of CFSs is vital for inhibiting cancer development. Using proteomics, SUMO2-PCNA was found to interact with the histone chaperones CAF1 and FACT, allowing for the accumulation of these factors at the replisome and a reduction in RNA Pol II within CFSs^124^. In this context, it was found that TRIM28 was the SUMO E3 ligase for PCNA and was required to prevent DNA breaks associated with transcription–replication conflicts at CFSs^110^ (Fig. 3a). The BRD protein TRIM28 is responsible for preventing transcription–replication conflicts to protect genome stability through its ability to SUMOylate PCNA. These activities by TRIM28 coordinate these processes through chromatin modulation at CFSs to ensure the resolution of any potential conflicts and the suppression of DNA break formation, which can be a threat to genome integrity.

## SMARCA4/BRG1

The BRD ATP-dependent chromatin remodeler SMARCA4 (BRG1) is involved in replication and transcription-associated DNA damage repair. Depletion of the ATPase BRG1 results in a genome-wide increase in R-loops and DNA breaks^4^ (Fig. 3c). Most of the R-loops identified were pre-existing structures found in control cells, but their frequency increased upon BRG1 knockdown. Given that the resultant DNA damage from BRG1 deficiency occurs preferentially in S-phase, the authors explored the involvement of BRG1 in resolving R-loop-mediated transcription–replication conflicts. Indeed, the loss of BRG1 reduced fork velocity and increased the association between replication forks and elongating RNA Pol II. These defects, including DNA damage, were suppressed by the expression of the R-loop resolver RNaseH1. BRG1 loss appeared to be epistatic with FANCD2, a factor involved in the Fanconi anemia pathway of DNA damage repair and a gene whose reduced expression also results in an increase in R-loop-mediated DNA damage. These findings suggest that BRG1 cooperates with the FA pathway to suppress and/or resolve R-loops involved in DNA damage that stems from transcription–replication conflicts (Fig. 3c). The BAF complex is one of several SWI/SNF ATP-dependent chromatin-remodeling complexes in mammals, and ARID1A is a core DNA-binding subunit of the BAF complex. Tsai S et al.^125^ discovered that loss of ARID1A induces transcription–replication conflicts and R-loop accumulation. Of note, an analysis of subunits of several SWI/SNF complexes revealed that not all promote R-loops and DNA damage when depleted, suggesting that specific SWI/SNF complexes have these functions. Finally, it has been reported that 20% of human cancers contain mutations or alterations in SWI/SNF complex-containing genes^126^. It is worth investigating whether tumors containing mutations in BRG1 or other SWI/SNF protein complex members display increased R-loops and DNA damage. These findings could reveal tumor-promoting roles for R-loop dysregulation and potential therapeutic strategies targeting R-loops and/or DNA damage pathways.

## Conclusion

While endogenous DNA damage contributes to tumorigenesis, identifying how this occurs through mechanistic studies has been challenging. A recent genome-wide sequencing study reported that endogenous DNA damage is the major source of genome instability and mutational signatures in specific cancers^8^. Future work must address the causes and consequences of endogenous DNA damage induction and identify which alterations in cells result in these changes and the ability of cells to be transformed in the presence of intrinsic DNA damage mechanisms. For BRD proteins, we reviewed several studies that point to defects in replication, including transcription–replication conflicts and R-loops, in BRD-deficient cells as potential instigators of mutations and tumorigenesis. BRD proteins also represent therapeutic targets in cancers, as they are mutated, misexpressed, and found as oncogenic fusion partners in various cancers^17,127,128^. Although these studies focused mainly on targeting transcriptional pathways involving BRD proteins, the studies discussed here suggest that additional targets such as R-loops and replication stress response pathways are worth consideration in the context of therapeutic strategies^129^.

**Fig. 4:**
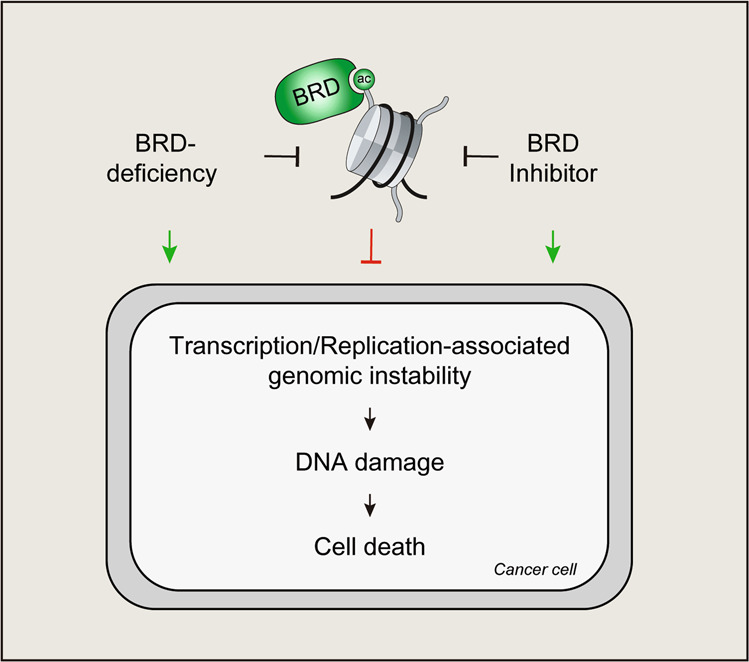
BRD proteins and their inhibitors in cancer. BRD protein deficiency or inhibition using small-molecule inhibitors can block normal function, resulting in induced transcription/replication-mediated genomic instability and cell death in cancer cells.

Large-scale proteomic studies have identified numerous BRD protein interactions with R-loops and replication forks^71,130^. Over half of BRD proteins also promote DSB repair, and many BRD proteins function in complex with other BRD proteins^14,131^. Given the involvement of BRD proteins in several genome integrity pathways and the complexity of their interactions, additional studies are needed to mechanistically identify how BRD protein loss or cancer-associated mutations affect these pathways. In addition, the development of new BRD inhibitors as anticancer therapies has outpaced our understanding of how BRD proteins contribute to genome integrity (Fig. 4). Determining how BRD proteins function in normal and cancer cells is essential not only for understanding the mechanisms involved in tumorigenesis but also for developing therapeutic strategies to target these proteins. One could imagine that BRD inhibitors could have deleterious consequences for genome integrity, similar to those for BET inhibitors, which could contribute to cellular toxicity and unwanted side effects (Fig. 4). Although much progress has been made in understanding endogenous DNA damage and how it promotes mutations^2^, additional work is needed to fully appreciate the contributions of BRD proteins and the inhibitors being developed to target them in influencing the regulation and production of endogenous DNA damage that contributes to mutagenesis, tumorigenesis, and therapeutic responses. In conclusion, given the intricate relationship between replication, transcription, and DNA repair, additional studies aimed at revealing how BRD chromatin reader proteins function within chromatin to coordinate these activities on DNA will provide deeper insights into the essential functions of BRD proteins as protectors of endogenous DNA damage that persistently threatens epigenome and genome integrity.

## References

[1] Lindahl T, Barnes DE (2000). Repair of endogenous DNA damage. Cold Spring Harb. Symp. Quant. Biol..

[2] Tubbs A, Nussenzweig A (2017). Endogenous DNA damage as a source of genomic instability in cancer. Cell.

[3] Lindahl T (1993). Instability and decay of the primary structure of DNA. Nature.

[4] Bayona-Feliu A, Barroso S, Munoz S, Aguilera A (2021). The SWI/SNF chromatin remodeling complex helps resolve R-loop-mediated transcription-replication conflicts. Nat. Genet..

[5] Haber JE (2015). Deciphering the DNA damage response. Cell.

[6] Martinez-Jimenez F (2020). A compendium of mutational cancer driver genes. Nat. Rev. Cancer.

[7] Bailey MH (2018). Comprehensive characterization of cancer driver genes and mutations. Cell.

[8] Alexandrov LB (2020). The repertoire of mutational signatures in human cancer. Nature.

[9] Davies H (2017). HRDetect is a predictor of BRCA1 and BRCA2 deficiency based on mutational signatures. Nat. Med..

[10] Zou X (2021). A systematic CRISPR screen defines mutational mechanisms underpinning signatures caused by replication errors and endogenous DNA damage. Nat. Cancer.

[11] Campbell BB (2017). Comprehensive analysis of hypermutation in human cancer. Cell.

[12] Kim JJ, Lee SY, Miller KM (2019). Preserving genome integrity and function: the DNA damage response and histone modifications. Crit. Rev. Biochem. Mol. Biol..

[13] Filippakopoulos P (2012). Histone recognition and large-scale structural analysis of the human bromodomain family. Cell.

[14] Kim JJ (2019). Systematic bromodomain protein screens identify homologous recombination and R-loop suppression pathways involved in genome integrity. Genes Dev..

[15] Gong F (2015). Screen identifies bromodomain protein ZMYND8 in chromatin recognition of transcription-associated DNA damage that promotes homologous recombination. Genes Dev..

[16] Stathis A, Bertoni F (2018). BET proteins as targets for anticancer treatment. Cancer Discov..

[17] Shorstova T, Foulkes WD, Witcher M (2021). Achieving clinical success with BET inhibitors as anti-cancer agents. Br. J. Cancer.

[18] Alqahtani A (2019). Bromodomain and extra-terminal motif inhibitors: a review of preclinical and clinical advances in cancer therapy. Future Sci. OA.

[19] Ameratunga M (2020). First-in-human Phase 1 open label study of the BET inhibitor ODM-207 in patients with selected solid tumours. Br. J. Cancer.

[20] Tsegay PS, Lai Y, Liu Y (2019). Replication stress and consequential instability of the genome and epigenome. Molecules.

[21] Tomasetti C, Vogelstein B (2015). Cancer etiology. Variation in cancer risk among tissues can be explained by the number of stem cell divisions. Science.

[22] Wilhelm T, Said M, Naim V (2020). DNA replication stress and chromosomal instability: dangerous liaisons. Genes (Basel).

[23] Gaillard H, Garcia-Muse T, Aguilera A (2015). Replication stress and cancer. Nat. Rev. Cancer.

[24] Li GM (2008). Mechanisms and functions of DNA mismatch repair. Cell Res..

[25] Abbas T (2021). The role of ubiquitination and SUMOylation in DNA replication. Curr. Issues Mol. Biol..

[26] Barbari SR, Shcherbakova PV (2017). Replicative DNA polymerase defects in human cancers: consequences, mechanisms, and implications for therapy. DNA Repair (Amst.).

[27] Bartkova J (2005). DNA damage response as a candidate anti-cancer barrier in early human tumorigenesis. Nature.

[28] Gorgoulis VG (2005). Activation of the DNA damage checkpoint and genomic instability in human precancerous lesions. Nature.

[29] Poot RA (2004). The Williams syndrome transcription factor interacts with PCNA to target chromatin remodelling by ISWI to replication foci. Nat. Cell Biol..

[30] Ribeyre C (2016). Nascent DNA proteomics reveals a chromatin remodeler required for topoisomerase I loading at replication forks. Cell Rep..

[31] Bhaskara S (2013). Histone deacetylases 1 and 2 maintain S-phase chromatin and DNA replication fork progression. Epigenetics Chromatin.

[32] Pommier Y (2009). DNA topoisomerase I inhibitors: chemistry, biology, and interfacial inhibition. Chem. Rev..

[33] Strumberg D (2000). Conversion of topoisomerase I cleavage complexes on the leading strand of ribosomal DNA into 5’-phosphorylated DNA double-strand breaks by replication runoff. Mol. Cell Biol..

[34] Goodman RH, Smolik S (2000). CBP/p300 in cell growth, transformation, and development. Genes Dev..

[35] Chan HM, La Thangue NB (2001). p300/CBP proteins: HATs for transcriptional bridges and scaffolds. J. Cell Sci..

[36] Kalkhoven E (2004). CBP and p300: HATs for different occasions. Biochem. Pharmacol..

[37] Stauffer D, Chang B, Huang J, Dunn A, Thayer M (2007). p300/CREB-binding protein interacts with ATR and is required for the DNA replication checkpoint. J. Biol. Chem..

[38] Cazzalini O (2014). CBP and p300 acetylate PCNA to link its degradation with nucleotide excision repair synthesis. Nucleic Acids Res..

[39] Mailand N, Gibbs-Seymour I, Bekker-Jensen S (2013). Regulation of PCNA-protein interactions for genome stability. Nat. Rev. Mol. Cell Biol..

[40] Moldovan GL, Pfander B, Jentsch S (2007). PCNA, the maestro of the replication fork. Cell.

[41] Boehm EM, Gildenberg MS, Washington MT (2016). The many roles of PCNA in eukaryotic DNA replication. Enzymes.

[42] Hasan S (2001). Regulation of human flap endonuclease-1 activity by acetylation through the transcriptional coactivator p300. Mol. Cell.

[43] Balakrishnan L, Stewart J, Polaczek P, Campbell JL, Bambara RA (2010). Acetylation of Dna2 endonuclease/helicase and flap endonuclease 1 by p300 promotes DNA stability by creating long flap intermediates. J. Biol. Chem..

[44] Yu BD, Hess JL, Horning SE, Brown GA, Korsmeyer SJ (1995). Altered Hox expression and segmental identity in Mll-mutant mice. Nature.

[45] Liu H, Cheng EH, Hsieh JJ (2007). Bimodal degradation of MLL by SCFSkp2 and APCCdc20 assures cell cycle execution: a critical regulatory circuit lost in leukemogenic MLL fusions. Genes Dev..

[46] Jude CD (2007). Unique and independent roles for MLL in adult hematopoietic stem cells and progenitors. Cell Stem Cell.

[47] Wang P (2009). Global analysis of H3K4 methylation defines MLL family member targets and points to a role for MLL1-mediated H3K4 methylation in the regulation of transcriptional initiation by RNA polymerase II. Mol. Cell Biol..

[48] Tyagi S, Chabes AL, Wysocka J, Herr W (2007). E2F activation of S phase promoters via association with HCF-1 and the MLL family of histone H3K4 methyltransferases. Mol. Cell.

[49] Milne TA (2005). Menin and MLL cooperatively regulate expression of cyclin-dependent kinase inhibitors. Proc. Natl. Acad. Sci. USA..

[50] Liu H (2010). Phosphorylation of MLL by ATR is required for execution of mammalian S-phase checkpoint. Nature.

[51] Ballabio E, Milne TA (2014). Epigenetic control of gene expression in leukemogenesis: cooperation between wild type MLL and MLL fusion proteins. Mol. Cell Oncol..

[52] Espana-Agusti J, Warren A, Chew SK, Adams DJ, Matakidou A (2017). Loss of PBRM1 rescues VHL dependent replication stress to promote renal carcinogenesis. Nat. Commun..

[53] Kurashima K (2020). SMARCA4 deficiency-associated heterochromatin induces intrinsic DNA replication stress and susceptibility to ATR inhibition in lung adenocarcinoma. NAR. Cancer.

[54] Cohen SM (2010). BRG1 co-localizes with DNA replication factors and is required for efficient replication fork progression. Nucleic Acids Res..

[55] Liu K, Luo Y, Lin FT, Lin WC (2004). TopBP1 recruits Brg1/Brm to repress E2F1-induced apoptosis, a novel pRb-independent and E2F1-specific control for cell survival. Genes Dev..

[56] Knudsen KE (2000). RB-dependent S-phase response to DNA damage. Mol. Cell Biol..

[57] Gupta M (2020). BRG1 loss predisposes lung cancers to replicative stress and ATR dependency. Cancer Res..

[58] Bell SP, Dutta A (2002). DNA replication in eukaryotic cells. Annu. Rev. Biochem..

[59] Diffley JF (2004). Regulation of early events in chromosome replication. Curr. Biol..

[60] Neelsen KJ (2013). Deregulated origin licensing leads to chromosomal breaks by rereplication of a gapped DNA template. Genes Dev..

[61] Williamson CT (2016). ATR inhibitors as a synthetic lethal therapy for tumours deficient in ARID1A. Nat. Commun..

[62] Alver RC, Chadha GS, Blow JJ (2014). The contribution of dormant origins to genome stability: from cell biology to human genetics. DNA Repair.

[63] Halazonetis TD, Gorgoulis VG, Bartek J (2008). An oncogene-induced DNA damage model for cancer development. Science.

[64] Kotsantis P, Petermann E, Boulton SJ (2018). Mechanisms of oncogene-induced replication stress: jigsaw falling into place. Cancer Discov..

[65] Di Micco R (2006). Oncogene-induced senescence is a DNA damage response triggered by DNA hyper-replication. Nature.

[66] Yan K (2016). The chromatin regulator BRPF3 preferentially activates the HBO1 acetyltransferase but is dispensable for mouse development and survival. J. Biol. Chem..

[67] Feng Y (2016). BRPF3-HBO1 regulates replication origin activation and histone H3K14 acetylation. EMBO J..

[68] Doyon Y (2006). ING tumor suppressor proteins are critical regulators of chromatin acetylation required for genome expression and perpetuation. Mol. Cell.

[69] Mandemaker IK (2018). DNA damage-induced replication stress results in PA200-proteasome-mediated degradation of acetylated histones. EMBO Rep..

[70] Donati B, Lorenzini E, Ciarrocchi A (2018). BRD4 and Cancer: going beyond transcriptional regulation. Mol. Cancer.

[71] Wessel SR, Mohni KN, Luzwick JW, Dungrawala H, Cortez D (2019). Functional analysis of the replication fork proteome identifies BET proteins as PCNA regulators. Cell Rep..

[72] Kang MS (2019). PCNA unloading is negatively regulated by BET proteins. Cell Rep..

[73] Lee KY (2010). Human ELG1 regulates the level of ubiquitinated proliferating cell nuclear antigen (PCNA) through Its interactions with PCNA and USP1. J. Biol. Chem..

[74] Lee KY, Fu H, Aladjem MI, Myung K (2013). ATAD5 regulates the lifespan of DNA replication factories by modulating PCNA level on the chromatin. J. Cell Biol..

[75] Kang MS (2019). Regulation of PCNA cycling on replicating DNA by RFC and RFC-like complexes. Nat. Commun..

[76] Bellaoui M (2003). Elg1 forms an alternative RFC complex important for DNA replication and genome integrity. EMBO J..

[77] Zhang J (2018). BRD4 facilitates replication stress-induced DNA damage response. Oncogene.

[78] Sansam CG (2018). A mechanism for epigenetic control of DNA replication. Genes Dev..

[79] Bai G (2020). HLTF promotes fork reversal, limiting replication stress resistance and preventing multiple mechanisms of unrestrained DNA synthesis. Mol. Cell.

[80] Kolinjivadi AM (2017). Smarcal1-mediated fork reversal triggers Mre11-dependent degradation of nascent DNA in the absence of Brca2 and stable Rad51 nucleofilaments. Mol. Cell.

[81] Poole LA, Cortez D (2017). Functions of SMARCAL1, ZRANB3, and HLTF in maintaining genome stability. Crit. Rev. Biochem. Mol. Biol..

[82] Vujanovic M (2017). Replication fork slowing and reversal upon DNA damage require PCNA polyubiquitination and ZRANB3 DNA translocase activity. Mol. Cell.

[83] Schlacher K (2011). Double-strand break repair-independent role for BRCA2 in blocking stalled replication fork degradation by MRE11. Cell.

[84] Schlacher K, Wu H, Jasin M (2012). A distinct replication fork protection pathway connects Fanconi anemia tumor suppressors to RAD51-BRCA1/2. Cancer Cell.

[85] Ray Chaudhuri A (2016). Replication fork stability confers chemoresistance in BRCA-deficient cells. Nature.

[86] Kim JJ (2020). PCAF-mediated histone acetylation promotes replication fork degradation by MRE11 and EXO1 in BRCA-deficient cells. Mol. Cell.

[87] Noordermeer SM, van Attikum H (2019). PARP inhibitor resistance: a tug-of-war in BRCA-mutated cells. Trends Cell Biol..

[88] Lemacon D (2017). MRE11 and EXO1 nucleases degrade reversed forks and elicit MUS81-dependent fork rescue in BRCA2-deficient cells. Nat. Commun..

[89] Thakar T, Moldovan GL (2021). The emerging determinants of replication fork stability. Nucleic Acids Res..

[90] Leuzzi G, Taglialatela A, Ciccia A (2020). HATtracting nucleases to stalled forks. Mol. Cell.

[91] Gomez-Gonzalez B, Aguilera A (2019). Transcription-mediated replication hindrance: a major driver of genome instability. Genes Dev..

[92] Hatchi E (2015). BRCA1 recruitment to transcriptional pause sites is required for R-loop-driven DNA damage repair. Mol. Cell.

[93] Bhatia V (2014). BRCA2 prevents R-loop accumulation and associates with TREX-2 mRNA export factor PCID2. Nature.

[94] Garcia-Rubio ML (2015). The Fanconi anemia pathway protects genome integrity from R-loops. PLoS Genet.

[95] Schwab RA (2015). The Fanconi anemia pathway maintains genome stability by coordinating replication and transcription. Mol. Cell.

[96] Winter GE (2017). BET bromodomain proteins function as master transcription elongation factors independent of CDK9 recruitment. Mol. Cell.

[97] Itzen F, Greifenberg AK, Bosken CA, Geyer M (2014). Brd4 activates P-TEFb for RNA polymerase II CTD phosphorylation. Nucleic Acids Res..

[98] Yang Z (2005). Recruitment of P-TEFb for stimulation of transcriptional elongation by the bromodomain protein Brd4. Mol. Cell.

[99] Edwards DS (2020). BRD4 prevents R-Loop formation and transcription-replication conflicts by ensuring efficient transcription elongation. Cell Rep..

[100] Lam FC (2020). BRD4 prevents the accumulation of R-loops and protects against transcription-replication collision events and DNA damage. Nat. Commun..

[101] Champoux JJ (2001). DNA topoisomerases: structure, function, and mechanism. Annu. Rev. Biochem..

[102] Baranello L (2016). RNA polymerase II regulates topoisomerase 1 activity to favor efficient transcription. Cell.

[103] Manzo SG (2018). DNA Topoisomerase I differentially modulates R-loops across the human genome. Genome Biol..

[104] Hamperl S, Cimprich KA (2016). Conflict resolution in the genome: how transcription and replication make it work. Cell.

[105] Bowry A, Piberger AL, Rojas P, Saponaro M, Petermann E (2018). BET inhibition induces HEXIM1- and RAD51-dependent conflicts between transcription and replication. Cell Rep..

[106] Wei L, Zhao X (2017). Roles of SUMO in replication initiation, progression, and termination. Adv. Exp. Med. Biol..

[107] Watts FZ (2006). Sumoylation of PCNA: wrestling with recombination at stalled replication forks. DNA Repair.

[108] Moldovan GL (2012). Inhibition of homologous recombination by the PCNA-interacting protein PARI. Mol. Cell.

[109] Gali H (2012). Role of SUMO modification of human PCNA at stalled replication fork. Nucleic Acids Res..

[110] Li M, Xu X, Chang CW, Liu Y (2020). TRIM28 functions as the SUMO E3 ligase for PCNA in prevention of transcription induced DNA breaks. Proc. Natl. Acad. Sci. USA..

[111] Aygun O, Svejstrup J, Liu Y (2008). A RECQ5-RNA polymerase II association identified by targeted proteomic analysis of human chromatin. Proc. Natl. Acad. Sci. USA.

[112] Aygun O (2009). Direct inhibition of RNA polymerase II transcription by RECQL5. J. Biol. Chem..

[113] Hu Y (2005). Recql5 and Blm RecQ DNA helicases have nonredundant roles in suppressing crossovers. Mol. Cell Biol..

[114] Hu Y (2007). RECQL5/Recql5 helicase regulates homologous recombination and suppresses tumor formation via disruption of Rad51 presynaptic filaments. Genes Dev..

[115] Li M, Pokharel S, Wang JT, Xu X, Liu Y (2015). RECQ5-dependent SUMOylation of DNA topoisomerase I prevents transcription-associated genome instability. Nat. Commun..

[116] Li M, Xu X, Liu Y (2011). The SET2-RPB1 interaction domain of human RECQ5 is important for transcription-associated genome stability. Mol. Cell. Biol..

[117] Saponaro M (2014). RECQL5 controls transcript elongation and suppresses genome instability associated with transcription stress. Cell.

[118] Sarni D, Kerem B (2016). The complex nature of fragile site plasticity and its importance in cancer. Curr. Opin. Cell Biol..

[119] Li S, Wu X (2020). Common fragile sites: protection and repair. Cell Biosci..

[120] Debatisse M, Le Tallec B, Letessier A, Dutrillaux B, Brison O (2012). Common fragile sites: mechanisms of instability revisited. Trends Genet.

[121] Hellman A (2002). A role for common fragile site induction in amplification of human oncogenes. Cancer Cell.

[122] Miller CT (2006). Genomic amplification of MET with boundaries within fragile site FRA7G and upregulation of MET pathways in esophageal adenocarcinoma. Oncogene.

[123] Glover TW, Wilson TE, Arlt MF (2017). Fragile sites in cancer: more than meets the eye. Nat. Rev. Cancer.

[124] Li M (2018). SUMO2 conjugation of PCNA facilitates chromatin remodeling to resolve transcription-replication conflicts. Nat. Commun..

[125] Tsai S (2021). ARID1A regulates R-loop associated DNA replication stress. PLoS Genet..

[126] Kadoch C (2013). Proteomic and bioinformatic analysis of mammalian SWI/SNF complexes identifies extensive roles in human malignancy. Nat. Genet..

[127] Fujisawa T, Filippakopoulos P (2017). Functions of bromodomain-containing proteins and their roles in homeostasis and cancer. Nat. Rev. Mol. Cell Biol..

[128] Perez-Salvia M, Esteller M (2017). Bromodomain inhibitors and cancer therapy: from structures to applications. Epigenetics.

[129] Lam FC, Kong YW, Yaffe MB (2020). Inducing DNA damage through R-loops to kill cancer cells. Mol. Cell. Oncol..

[130] Cristini A, Groh M, Kristiansen MS, Gromak N (2018). RNA/DNA hybrid interactome identifies DXH9 as a molecular player in transcriptional termination and R-loop-associated DNA damage. Cell Rep..

[131] Chiu LY, Gong F, Miller KM (2017). Bromodomain proteins: repairing DNA damage within chromatin. Philos. Trans. R. Soc. Lond. B. Biol. Sci..

